# The measurement reliability and equivalence of print versus online versions of the Youth Activity Profile

**DOI:** 10.1371/journal.pone.0312254

**Published:** 2025-01-24

**Authors:** Yang Bai, Philip M. Dixon, Pedro F. Saint-Maurice, Paul R. Hibbing, Gabriella M. McLoughlin, Michael Pereira da Silva, Gregory J. Welk

**Affiliations:** 1 Department of Health and Kinesiology, University of Utah, Salt Lake City, Utah, United States of America; 2 Department of Statistics, Iowa State University, Ames, Iowa, United States of America; 3 Champalimaud Foundation, Lisbon, Portugal; 4 Department of Kinesiology and Nutrition, University of Illinois Chicago, Chicago, Illinois, United States of America; 5 Department of Social and Behavioral Sciences, Temple University, Philadelphia, Pennsylvania, United States of America; 6 Faculty of Medicine, Federal University of Rio Grande, Rio Grande, Rio Grande do Sul, Brazil; 7 Department of Kinesiology, Iowa State University, Ames, Iowa, United States of America; UBC: The University of British Columbia, CANADA

## Abstract

**Purpose:**

The Youth Activity Profile (YAP) is a 7-day self-report designed to quantify physical activity and sedentary behaviors among youth. This study evaluated the reliability of the online version of the YAP and equivalence with the paper-based version.

**Method:**

A total of 2,490 participants from 17 schools in Iowa and Texas completed the YAP. Each classroom was randomly assigned to complete either the print or online version twice. A variance components model was used to assess the test-retest reliability and equivalence testing was also applied.

**Results:**

Both paper and online versions had similar reliability for the PA estimates in school (ICC_print_ = 0.69–0.91; ICC_online_ = 0.54–0.84), at home (ICC_print_ = 0.72–0.83; ICC_online_ = 0.64–0.94), PA at weekend (ICC_print_ = 0.33–0.72; ICC_online_ = 0.39–0.70), and SB (ICC_print_ = 0.69–0.90; ICC_online_ = 0.66–0.80). The two versions were statistically equivalent for most YAP items except for recess.

**Conclusion:**

The online YAP appears to be a reliable assessment of physical activity and sedentary behavior in youth populations.

## Introduction

The development of accurate methods to assess physical activity (PA) and sedentary behavior (SB) remain a high priority for public health research [1, 2]. While there are many advantages of monitor-based measures, challenges remain for applying them in population studies or for surveillance applications and unique challenges exist for youth [3–5]. Monitor-based measures are also more difficult to use by practitioner such as physical education teachers in school settings to assess youth behaviors due to the cost, complexity, and time required to process and interpret data [6, 7]. Thus, it is important to continue to advance research on the utility of report-based measures [8, 9].

The Youth Activity Profile (YAP) is a 15-item self-report instrument with time-based structure designed to capture PA and sedentary behavior over a 7 days period. Regarding the PA measure, the YAP provides information of PA practiced in different contexts such as at school, out of school, and during the weekend. A unique feature of the YAP is that it has been calibrated against monitor-based methods to provide accurate group-level estimates of PA and SB [10]. The YAP has recently been adapted from a print-based to an online version and a recent study supported the validity of new algorithms [11]. The transition to online survey formats provides many advantages over traditional print formats [12, 13]. Online surveys reduce the potential for data entry errors by eliminating the need to transfer the data from paper to computer [14]. Additionally, the time- and cost-effectiveness of online surveys overcome two of the biggest barriers to administering the surveys in school settings [15, 16]. The latter benefits were driving factors in adaptation of the YAP to online format, since it was originally designed for assessment and promotion of PA in school settings [17].

Online and paper survey can differ in several ways that may impact the responses obtained. One key factor is the mode of administration, with research suggesting that internet-based surveys may yield lower response rates but higher response quality than traditional paper surveys. This difference in mode of administration can affect how respondents perceive and interact with the survey [18, 19]. Another factor is visual layout, as the appearance of the survey may influence how respondents interpret and answer questions [20]. For example, an online survey may have larger fonts, more white space, and images, which were utilized in the YAP online platform. Response options may differ between online and paper surveys, with electronic surveys offering more flexibility and potentially reducing errors [21]. YAP surveys includes drop-down menus, skip patterns, and branching logic, which are not possible on paper surveys. This can affect the types of responses that are given. Technology used to administer the survey can also affect response quality, due to technical issues such as slow internet connections, compatibility issues, or device failures [22, 23]. Research showed that younger respondents may be more likely to complete an online survey, which is the targeting population for YAP. Respondent motivation may be a key factor, as the convenience and anonymity of online surveys may increase participation but also lead to less thoughtful or accurate responses [24]. Overall, these mechanisms can lead to differences in how respondents perceive and interact with online and paper surveys, which can affect the quality and completeness of the data collected.

A well-established tenet of research is that reliability is a pre-requisite for validity, but few studies have evaluated the reliability of report-based measures of physical activity due to the complexity of the behavior. Previous research by our team has reported good group-level agreement between the YAP and linked estimates from a monitor (i.e. validity). However, to reduce error and improve precision it is important to fully understand the psychometrics of the instrument. It is critical to understand the reliability in different settings (school and home) as well as for discrete periods. Because the instrument has been re-developed for use online it is also important to also directly evaluate reliability of print and online formats to ensure that the implementation method doesn’t influence the results. A systematic review of youth measures specifically highlighted the need to examine both reliability and validity to fully evaluate the measurement characteristics of report-based instruments [25]. Recent research has examined the reliability of the printed version of the YAP in a sample of Spanish youth [26]. However, it is crucial to assess the newer online format using a larger and more diverse sample. Thus, the present study was intentionally designed to evaluate and compare the test-retest reliability and equivalence from both the print and online versions of YAP. The systematic evaluation of individual items provides new insights since reliability may vary for periods during school or outside of school time. By comparing responses from multiple sites and across multiple age groups, the study also provides novel insights about how measurement characteristics may vary across these different dimensions.

## Method

### Study design and sample

Data were collected between Spring of 2014 to Fall 2015 from 17 schools located in Texas (Dallas area) and Iowa (Des Moines area). The data were collected from large, ethnically diverse districts in the two states to provide generalizable information. Schools were recruited with help from the District PE coordinators to ensure there was a balance of both low and high SES schools from Elementary, Middle and High Schools in the District. The data collection was conducted with assistance from the PE teachers using intact classes to ensure that the survey administration were reflective of how the YAP would be used in school. Eight schools were recruited from Dallas: 3 elementary, 2 middle, 1 high school, 1 with combined grades for elementary and middle school, and 1 with combined grades from all three levels. Three grades of 5th, 7th, and 9th were selected to represent elementary, middle, and high school students. Nine schools were recruited from Iowa: 3 elementary, 3 middle, 2 high school and 1 with combined grades for elementary and middle school. Across sites, 2,489 participants from which 1,227 boys and 1,261 girls (1 participant did not reveal the sex) completed the YAP twice each. Students were randomly assigned to complete either the paper version or online version. Each took their assigned version of the YAP twice, one week apart. The study protocol was approved by Iowa State University Institutional Review Board and parents provided informed consent for their child to participate and children completed an assent form. The average of the two trials data were used to compute for version, site, age group, and sex. The breakdown of sample size by site, format, and grade level were summarized in Table 1.

**Table 1:** Mean and standard deviation of four YAP segments separate for trial, version, site, age group, and sex.

	PA in School		PA out of School		Weekend		SED	
	N	Mean (SD)	N	Mean (SD)	N	Mean (SD)	N	Mean (SD)
Overall	2489	2.74 (.94)	2483	3.29 (1.13)	2490	3.26 (1.12)	2477	2.63 (.77)
	Print							
**Trial**								
1	693	2.69 (.92)	690	3.20 (1.16)	691	3.18 (1.14)	693	2.71 (.77)
2	591	2.62 (.95)	588	3.16 (1.15)	591	3.15 (1.13)	584	2.65 (.79)
**Site**								
Iowa	550	2.53 (.90)	547	3.12 (1.17)	547	3.08 (1.16)	551	2.67 (.80)
Texas	733	2.75 (.95)	731	3.22 (1.15)	735	3.23 (1.11)	726	2.70 (.76)
**Age group**								
Elementary	462	2.93 (.90)	460	3.38 (1.15)	465	3.30 (1.11)	456	2.50 (.85)
Middle	501	2.57 (.93)	500	3.13 (1.11)	500	3.15 (1.13)	502	2.79 (.75)
High	320	2.40 (.89)	318	2.96 (1.19)	317	3.00 (1.17)	319	2.79 (.65)
**Sex**								
Boys	628	2.75 (.93)	628	3.20 (1.15)	629	3.24 (1.13)	624	2.69 (.75)
Girls	653	2.56 (.93)	648	3.15 (1.17)	651	3.09 (1.14)	651	2.68 (.81)
	Online							
**Trial**								
1	753	2.84 (.93)	752	3.39 (1.08)	755	3.33 (1.11)	752	2.62 (.76)
2	458	2.78 (.94)	458	3.41 (1.07)	458	3.43 (1.10)	453	2.51 (.75)
**Site**								
Iowa	572	2.83 (.94)	571	3.39 (1.09)	573	3.32 (1.16)	567	2.54 (.77)
Texas	646	2.82 (.93)	646	3.42 (1.06)	647	3.42 (1.06)	645	2.60 (.76)
**Age group**								
Elementary	523	3.16 (.89)	521	3.60 (1.03)	524	3.53 (1.05)	520	2.41 (.80)
Middle	447	2.66 (.88)	447	3.32 (1.01)	447	3.28 (1.12)	443	2.68 (.72)
High	248	2.39 (.86)	249	3.12 (1.19)	249	3.20 (1.16)	249	2.74 (.71)
**Sex**								
Boys	604	2.85 (.94)	605	3.47 (1.06)	605	3.53 (1.09)	600	2.61 (.75)
Girls	614	2.80 (.93)	612	3.33 (1.08)	615	3.21 (1.10)	612	2.54 (.78)

Note: PA, Physical Activity; SED, Sedentary Behavior; SD, Standard Deviation; all, the average of all 15 YAP items. Note: SD, Standard Deviation; all, the average of all 15 YAP items.

### Description of the YAP and administration procedures

The YAP comprises 15 total items each scored on a 5-point scale designed to capture four components in youth behaviors: 1) PA in school, 2) PA out of school, 3) PA over the weekend, and 4) SB [17, 27]. The first five items assess PA in school by asking about behavior in different school time periods (transportation to school, PE, recess, lunch and transportation back from school). Questions included items such as “During lunch break, how often were you moving around, walking or playing?” The next five questions asked about PA levels out of school. Three questions asked how many days they were active for at least 10 minutes before school, after school, and weeknights. Response options ranged from no days to 4–5 days as the highest option. Two additional questions asked more specifically about the length of time they spend on PA on Saturday and Sunday. The last five items asked about time spent in different sedentary behaviors (TV time, video games, computer time, Phone/Text time and overall). The print and online versions of the YAP were exactly the same regarding the content with the exception of additional images/figures and colored background in the online version (https://www.youthactivityprofile.org/demo). Students from both the online and paper groups received the same explanation.

Prior to survey completion, the researchers explained how to answer the questions and asked the students to recall their past seven days’ PA and SB. The explanations and included definitions (e.g. to clarify the meaning of “sedentary” or “moderate” for younger children) with examples given as needed. Students were guided through the assessment as needed but it was designed to be completed individually without requiring help from teachers. Students completing the online version were able to select their school by clicking their school name in a dropdown list. They were asked to provide some basic information about their sex, school level (elementary, middle or high school), and grade (from 3rd to 12th). Similar fields were included on the print version. The administration of both versions took 20 minutes on average. The students answered both print and online versions at school following the same procedures.

### Data processing and analysis

The data from the print version was entered by a research assistant and the online version data were downloaded from the YAP hosting server. All questions were scored in a range of 1 to 5. Higher scores indicated higher participation in PA or SB. The same coding scheme was used for print and online YAP.

The 15 YAP items were aggregated according to the general processing guidelines [27] to obtain indicators that capture PA in school (item 1–5), PA out of school (item 6–8), PA on weekends (item 9–10), and SB (item 11–15) (Box 1).

**Box 1. Youth Activity Profile scores.**

**Table:** 

	**YAP scores**
PA at school	Average of scores from questions 1 to 5
PA out of school	Average of scores from questions 6 to 8
PA on weekends	Average of scores from questions 9 and 10
Overall PA	Average of scores from questions 1 to 10
Sedentary behavior	Average of scores from questions 11 to 15

PA: physical activity

The four YAP segments were summarized (mean ± SD) separately for trial, version, site, age group, and sex. Intraclass correlation coefficients (ICC) and standard deviations were used to assess the test-retest reliability. The following ICC cut-off points were used to evaluate the level of reliability: ICC<0,50: poor reliability; ICC between 0.50 and 0.75: moderate reliability; ICC between 0.75 and 0.90: good reliability and ICC>0.90: excellent reliability [28].

To compute the ICC, a variance components model was fit to multiple YAP scores reported by the same individual. The model included random effects for school, individual within school, and observation within individual. This model was fit using SAS proc mixed, version 9.4. Variance components were estimated for each combination of activity segment (in school, out of school, weekend, and SB) and grade level because the variation between individuals was expected to differ between segments and potentially between grade levels. ICC were calculated from the estimated variance components as: $ICC=\frac{\sigma^{2}school+\sigma^{2}individual}{\sigma^{2}school+\sigma^{2}individual+\sigma^{2}observation}$ [29]. The standard deviation between repeated measurements on the same individual was estimated as $\sqrt{\sigma^{2}observation}$. This provides an absolute measure of repeatability [30]. Higher values of that standard deviation indicate lower repeatability between the two observations on the same individual. To compare repeatability between the online and print versions, we fit the variance components model separately for the two versions. To compare repeatability between IA and TX, we fit the variance components model separately to individuals in IA and individuals in TX.

Equivalence between the print and online YAP versions was assessed using the two-one-sided-tests (TOST) method and visualized using 90% confidence intervals for the ratio of the means for the two versions [31]. The 90% confidence interval for the ratio of online / print YAP was computed using Fieller’s theorem [32]. Sample averages and their standard errors were estimated using a mixed model with random effects to account for variation between schools, between participants within a school, and trials within a participant (the error variance). When the 90% confidence interval falls entirely inside the equivalence bounds, the p-value from a TOST has a p-value less than 5%. We defined the two versions to be equivalent if the ratio (online/print) was between 0.8 and 1.25 as it is a common practice adopted by Food and Drug Administration [33]. The analysis was repeated for each of the 15 YAP questions and five aggregated YAP summaries (i.e., activity at school, activity out of school, activity on the weekend, total activity, and total sedentary behavior).

## Results

Descriptive results were provided for YAP measures in Table 1. The YAP responses indicated lower levels of PA and higher levels of SB for older participants compared to younger. These patterns were evident for individual YAP summary segments (PA in school, PA before school, PA after school, PA at weekday evening). Boys reported more PA than girls, but SB was similar for both. Iowa participants reported more PA and slightly more SB than Texas participants. When comparing the two versions, students tended to report more PA and SB from the online tool than with the print version.

Table 2 shows ICCs for test-retest reliability. The two versions had all ICCs indicating a moderate reliability. The two versions showed similar reliability for PA in school (ICC_print_ = 0.69–0.91; ICC_online_ = 0.54–0.84), PA out of School (ICC_print_ = 0.72–0.83; ICC_online_ = 0.64–0.94), PA at weekend (ICC_print_ = 0.33–0.72; ICC_online_ = 0.39–0.70), and SB (ICC_print_ = 0.69–0.90; ICC_online_ = 0.66–0.80). The only poor reliability was found for weekend PA ICC for elementary schools (ICC = 0.33–0.39). Comparisons by region revealed similar reliability estimates across the four different components between Iowa and Texas. YAP completed by high schoolers had a higher ICC for PA in school and at home compared to surveys completed by elementary and middle schoolers.

**Table 2:** Intraclass correlation of YAP overall and separately for version, site, age group, and sex.

	PA in School	PA out of School	Weekend	SED
Overall	0.77	0.78	0.54	0.77
Print				
**Site**				
Iowa	0.79	0.74	0.47	0.90
Texas	0.81	0.81	0.56	0.71
**Age group**				
Elementary	0.69	0.79	0.33	0.78
Middle	0.81	0.76	0.72	0.83
High	0.91	0.81	0.50	0.79
**Sex**				
Boys	0.85	0.83	0.51	0.69
Girls	0.74	0.72	0.52	0.86
Online				
**Site**				
Iowa	0.75	0.76	0.48	0.66
Texas	0.70	0.77	0.57	0.80
**Age group**				
Elementary	0.76	0.64	0.39	0.76
Middle	0.54	0.73	0.57	0.73
High	0.84	0.94	0.70	0.72
**Sex**				
Boys	0.60	0.70	0.47	0.66
Girls	0.82	0.83	0.52	0.80

Note: PA, Physical Activity; SED, Sedentary Behavior.

**Fig 1:**
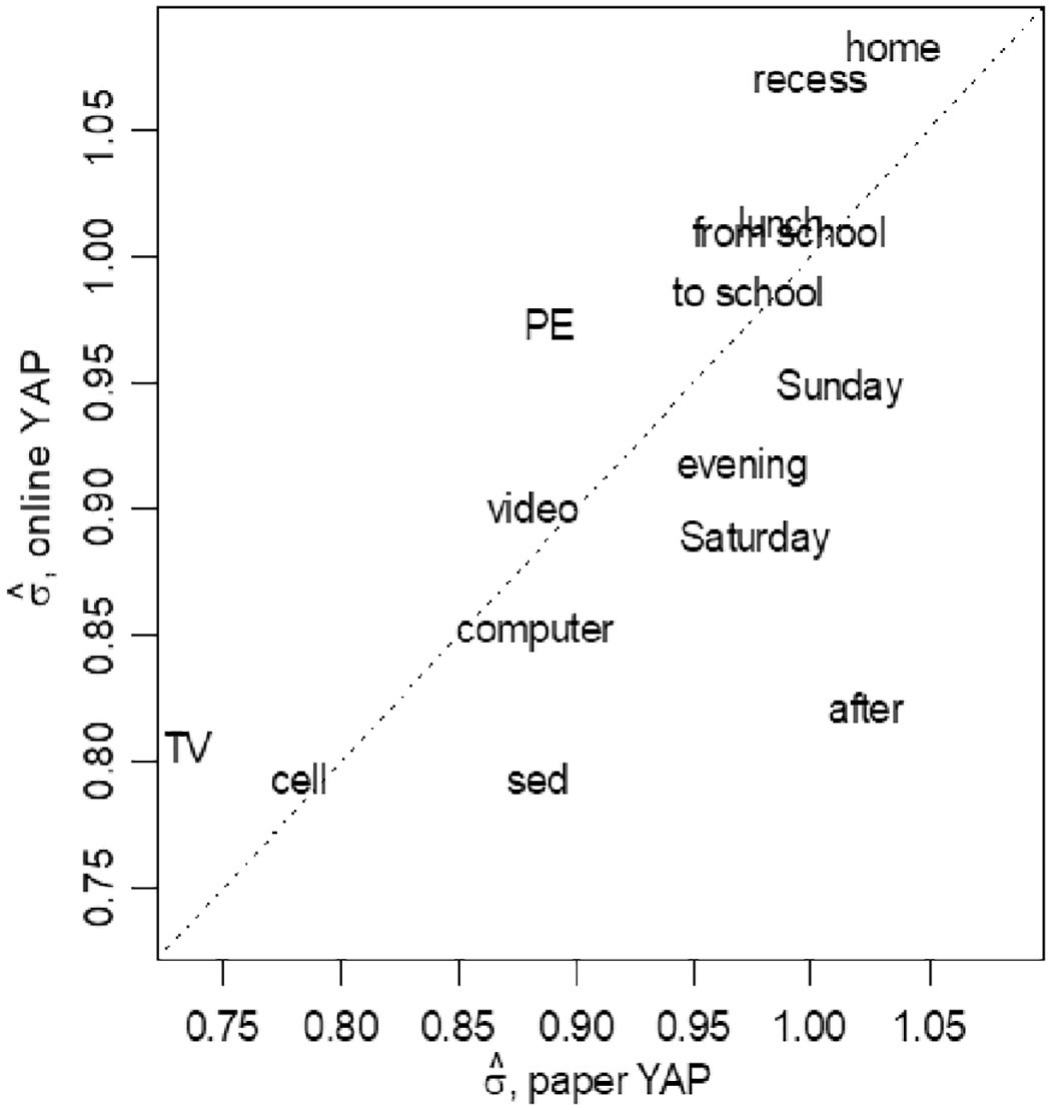
Standard deviation of repeated observations for print and online versions of 15 Youth Activity Profile items.

Standard deviations were similar for the online and print versions of each YAP item (Fig 1). Six items (i.e., Use of cellphone, Use of Computer, Use of video game, PA during lunch, PA to/from school) had similar standard deviation in both versions. The online version had higher standard deviations than the print version for 4 items (Time spent on TV, PA during PE, PA during recess and PE before school), but the differences were within 0.1. Higher values of that standard deviation showing the data is widely spread which indicates lower repeatability between the two observations on the same individual. There was no cut-off for standard deviation interpretation. We arbitrarily used 0.1 so that would be interpreted as 10% difference between print and online version of YAP in standard deviation. The print version had higher standard deviation than the online version for the rest of the 5 items. These differences were within 0.1 for four items (i.e., overall sedentary time, PA on Saturday and Sunday, PA in the evening), but the online version had much better reliability (lower standard deviations) for PA after school (standard deviation = 0.82) than the print version (standard deviation = 1.01). While strict measurement error between two assessments in the same week may not capture true test-retest reliability, the current of approach of recruiting two separate samples to independently complete either print or online version in two weeks allows researchers to understand both measurement error and natural variability in responses over time.

**Fig 2:**
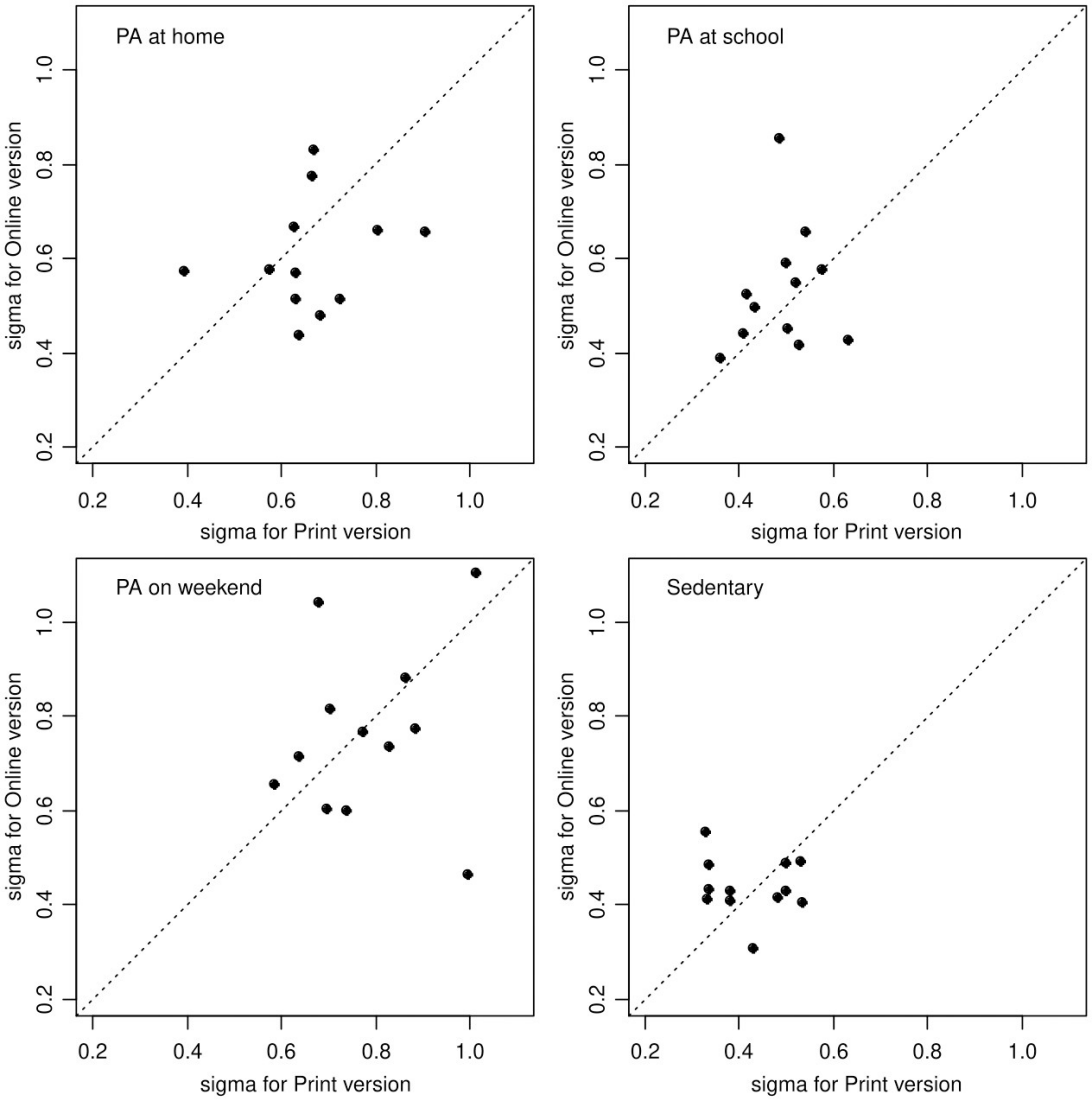
Standard deviation of repeated observations for print and online versions of Youth Activity Profile separately for major segments of the day. Note: PA, Physical Activity.

Fig 2 depicts the standard deviations of repeated measures for the print and online YAP items. Roughly similar patterns are seen when repeatability standard deviations are computed for subgroups of participants classified by site, age group, and sex. Fig 2 shows these patterns for 4 summary components of the YAP: PA at home, PA at school that combines, PA on weekend, that combines and total sedentary. There is a larger uncertainty in each estimated variance component because each subgroup has between 23 and 109 participants compared to 746 or 756 for the overall data set. At the subgroup level, there is no evidence of a difference in repeatability between the two versions (sign test, exact value p-value = 0.15). Repeatability of the total sedentary score is generally highest (smallest SDs) than that for either PA at home or PA at school. Repeatability for PA on the weekend is the lowest.

**Fig 3:**
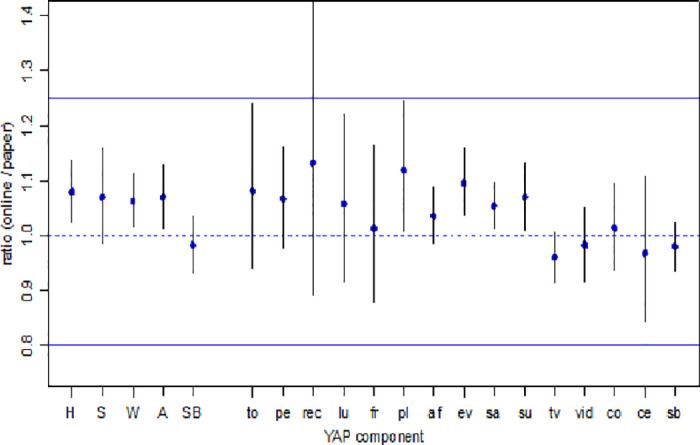
Estimated ratios (online / print mean YAP score) and 90% confidence intervals for each of the 5 aggregated YAP scores and the 15 individual YAP questions. Note: The solid horizontal lines are the equivalence bounds, i.e. the ratio is between 0.8 and 1.25; the dashed horizontal line is at a ratio of 1. The aggregated YAP scores are home (H), school (S), weekend (W), total activity (A), and total sedentary behavior (SB). The individual YAP questions are transportation to school (to), physical education (pe), recess (rec), lunch (lu), transportation from school (fr), play at home (pl), after school (af), evening (ev), Saturday (sa), Sunday (su), TV, video games (vid), computer (co), cell phone (ce), and overall sedentary behavior (sb).

Fig 3 shows the 90% confidence intervals for the 5 aggregated YAP categories and 15 YAP questions. Online and print version of YAP were statistically equivalent in all 5 aggregated YAP categories and 14 out of 15 YAP questions as the 90% confidence interval for the ratio of online YAP / print YAP falls entirely inside the 0.8 to 1.25 equivalence zones. The only item that was not equivalent between the two modules is the recess question. In general, the 5 aggregated categories had narrower 90% confidence interval than individual item indicating better measurement agreement between the online and print versions of YAP used to estimate the overall PA and SB.

## Discussion

The current study systematically evaluated the measurement characteristics of the new online version of the YAP by making direct comparisons to the traditional print version. The results document that assessments from the online version were statistically equivalent to those from the print version. The study revealed that both versions had similar reliability for PA during weekend and sedentary behaviors while print version had slightly higher reliability for PA in school and at home. Most of the YAP items had similar reliability for both versions.

It is a common research interest in surveillance studies to examine sex and age differences in PA levels [34], but research to date has rarely systematically evaluated reliability of PA instruments by these same dimensions. The study did not reveal any sex differences in reliability of recalling PA regardless of print or online version of YAP. However, differences in reliability were observed by age. It is not surprising that the older kids recall the PA and sedentary behaviors more reliable over time since they might engage in more structured exercise, have more consistent schedule, and increased capacity to recall their behaviors within a particular place and moment in time [35]. The structured exercise is easier to be recalled and it provides a more consistent activity pattern resulting in higher test-retest reliability.

We also found that the reliability varied across the various individual items, with sedentary items having higher reliability and several PA-related items with notably lower repeated reliability (i.e., PA during lunch, recess, transportation to/from school, and PA before school). The sporadic and intermittent nature of children’s PA limits their ability to recall the details of PA especially during unstructured play times such as recess and lunch break [36]. In contrast, some of the sedentary behaviors like TV watching or videogame playing might occur in longer duration and at certain time of the day especially for families with ‘screen time rules’, with which could ease the recalling of sedentary behaviors [37]. The interpretation of lunch or recess items in surveillance studies using YAP in elementary school should be cautious but it is still important to include them at school setting for education purpose.

Relatively few studies have directly compared the measurement characteristics and properties of print and online instruments, but our results agree with a previous investigation showing equivalence between the two formats [38]. Several studies have compared outcomes from mixed mode survey administrations [39, 40]. Differences in observed rates of health behaviors between print and online tools have been reported due to differences in response characteristics of the different formats [40]. Other studies have compared the usability of print and online tools by examining the time to complete the questionnaire, frequency of asking for help, error count, and performance score [41]. Higher user feasibility with web-based questionnaires was observed among older participants (i.e., 4th or 5th graders) and children who had a PC at home. The authors reported that using the scroll bar was the most common difficulty in completing the questionnaire and suggested that age/grade level and familiarity with technology may be important considerations when evaluating web-based tools designed for children. Acceptable reliability was observed in all age groups in our study and familiarity with computers was not noted as a barrier in the use of the YAP. A recent study directly compared the test–retest reliability and construct validity of an online and paper administered Physical Activity Neighborhood Environment Scale in Canadian adults and also found reliable overall agreement between the versions [42]. Our study along with Frehlich et al. study contributed to the PA and public health discipline by supporting that online survey could provide reliable data as the traditional print survey.

With the increasing emphasis on Comprehensive School PA Programs (CSPAP) [43, 44], it is particularly important to develop tools that can be efficiently deployed in school settings [45]. Self-report tools offer advantages since they are better suited to capturing context and personal reflection. Advantages of employing PA assessments within physical education have been previously described [7], but efficient implementation strategies are needed to enable use by teachers. The online administering mode of the YAP provides for a more effective implementation and the opportunity for youth to receive immediate feedback on their behavioral patterns. The physical educators could use the feedback to provide physical literacy education and self-monitoring skills to the students, especially for elementary students. It will also allow youth to set personal PA goals based on their individual preferences and capabilities. Regularly administer the survey to track progress towards these goals could provide feedback and encouragement to the participants. Teachers could incorporate educational content related to PA and health into the self-report survey administering by providing brief instructional messages, tips, and resources that aim to enhance participants’ knowledge and understanding of the importance of PA. The YAP platform is managed by teachers or school personnel who can upload class rosters, set-up personal student ID’s and see aggregated summary reports. It is designed to enable full administrative access to teachers and school administrators, including access to summarized group reports. Thus, the online YAP survey overcomes barriers associated with print surveys and provides a pedagogically sound approach to capture data on youth in schools.

A major strength of our study is that we examined the age and sex difference in measurement equivalence by recruiting a large sample of youth from elementary, middle, and high schools from two different states. Previous work by collaborators in Spain documented the feasibility and reliability in Spain [26], but the present study documents the utility of the online version and employed a larger sample and a more sophisticated analytic approach to capture item-specific data. Despite the strengths of this study, some limitations must be discussed to discern future directions for research. First, although it is important to establish the reliability of measurement tools like the YAP, it is not possible to assess true test and re-test reliability based on the variability between two measurements of the same individual in the same week, i.e., strict measurement error. We recognize that the outcomes do not reflect a traditional evaluation of test-retest reliability, but the standard deviation between weeks that we report here includes both the strict measurement error and the individual variability in activity from week to week. When the goal is to estimate average PA over a month or a year, the variability between weeks is important [46, 47]. The standard deviations reported here include that variation, at least over short time periods. This method has not been widely used in PA measurement field but utilizing two independent samples to complete either print or online version or using a cross over design in other fields [48, 49]. Conducting a test-retest reliability study using two random samples to compare print or online version PA surveys has important practical applications. It ensures consistency across formats, validating that results from either method are equivalent and reliable. This allows researchers and institutions to confidently use either format, knowing that the data’s integrity remains intact. This is crucial for estimating average PA over extended periods, where week-to-week variability is meaningful. The method accounts for these variations, making it particularly relevant when transitioning between survey formats. Second, although data were gathered from children in two states (Iowa and Texas), the results may not generalize across the United States. A more nationally representative sample is necessary to enhance the potential of the YAP and implications for surveillance. Future work with such a large, representative sample is therefore warranted.

## Conclusions

The purpose of this study was to assess the reliability and equivalence of the online YAP tool when compared to the traditional print version through a test-retest design. Findings showed acceptable reliability and strong equivalence, particularly for older age groups. Similar YAP reliability was found in boys and girls. Online tools, such as the YAP, may become more crucial as schools are encouraged to develop CSPAP or similar programs, and serve as both an assessment and instructional tool to help enhance the PA behaviors in youth. Further investigation is needed with larger and more diverse samples, to understand the potential of the YAP as a surveillance tool and enhance the public health relevance of online assessment as a means to enhance health behaviors in youth.

## Supporting information

S1 Data (XLSX)
